# Notes and News

**Published:** 1902-11-01

**Authors:** 


### NOTES AND NEWS.

The Edinburgh Public Health Committee have issued a circular to the medical profession asking them to co-operate by notifying all cases of phthisis.

In Germany medical students number about a quarter of all the students attending the universities. Many commence their studies before the age of 18.

The Eleventh International Congress of Hygiene and Demography will be held at Brussels in September 1903, under the patronage of the King of the Belgians.

The course of lectures at the Brompton Hospital for Consumption, in connection with the post-graduate school of the Associated Schools of Medicine in the Metropolis, commenced on Wednesday at 3 p.m., and will be continued throughout the session.

Last week the Princess Christian laid the foundation stone of the new Hampstead Hospital. After the ceremony the Princess received purses containing a collective sum of nearly £15,000. The new hospital is intended for 50 patients.

The Annual Medical Service inaugurated by the Guild of St. Luke took place on the 22nd inst. at St. Paul's Cathedral. The Bishop of Kensington preached the sermon, and the choral music was performed by a choir of 250.

Mr. Alban Doran will deliver the Harveian lectures of the Harveian Society on the first three Thursdays in November, at the Stafford Rooms, Tichborne Street, Edgware Road, at 8.30 p.m. He takes for his subject Uterine Fibroids, considered from a clinical and surgical standpoint.

At the meeting of medical men connected with St. George's Hospital, for the purpose of improving the financial position of the hospital, an able statement was made by Mr. Timothy Holmes. This was followed by the carrying of a resolution by those present, to do what they could to bring the needs of St. George's Hospital before the public.

The Dean and Chapter of Westminster have forwarded to the Lord Mayor £200 in lieu of the usual offertory in the Abbey for the Hospital Sunday Fund. The Abbey was closed for the Coronation preparations on Hospital Sunday, and the contribution is made from the money paid by the public for viewing the Abbey after the Coronation was over.

The *Guardian* for the present week publishes an article on "Medical Education." It is a useful statement of fact, containing all the information serviceable to parents considering a medical career for their sons. But it is a pity that the article did not appear a few months earlier. Before the next session of the schools it may be overlooked.

Portrush, in Ireland, is to have a cottage hospital. A local resident, Miss Reynolds, has offered a building and site for the purpose. The institution, when altered to the requirements of a hospital, will contain six rooms for patients. This will provide accommodation for accident and general cases and private wards. About £300 of the £500 to complete the work of alteration is still needed.

The committee of the West Suffolk Hospital at Bury St. Edmunds have devoted a large legacy, recently received, upon some much-needed improvements in the interior of the hospital. These include an installation of electric light throughout the building, the improvement of the nurses' sleeping apartments, a large new operating theatre at a cost of £2,500, and a large recreation room for the nurses has been built and furnished.

It is not generally known that the British Home for Incurables has lost its able secretary, Mr. R. Gofton Salmond. Mr. Salmond was only 51 years of age. He held his post at the Home for Incurables for twenty-one years, during which time he saw the institution increase greatly in importance, usefulness, and prosperity. Mr. Salmond may be described as having a distinct personality. The task of replacing him will not be an easy one. Administrative ability, energy, experience, high personal character, and considerable address are required for the important post rendered vacant by Mr. Salmond's death.

Sir Henry Burdett addressed a crowded meeting at Croydon last week in aid of the League of Mercy, Sir William Treloar being in the chair. Sir Henry Burdett exhibited his series of living pictures from the hospitals, and explained that the object of the League was to secure the co-operation of all residents in London and the surrounding districts who realise the privilege of rendering a measure of personal service to the sick, and to devote three days in each year to the work of the League. The result of the meeting at Croydon was full of promise, as the whole of the large audience practically joined the League by adopting the scheme of the family gift. Other meetings are fixed for October 29th at Plumstead, October 31st at Norwood Technical Institute, West Norwood, at 5 p.m., and on November 14th at Twickenham Town Hall.

